# Comparative Ethnomedicinal Knowledge and Medicinal Plant Use among Indigenous Communities in Southwestern China and Northern Pakistan

**DOI:** 10.12669/pjms.42.7.19776

**Published:** 2026-07

**Authors:** Zhang Tong, Dong Jiying, Qu Qiumei

**Affiliations:** 1 Zhang Tong Lecturer, College of Humanities, Heilongjiang Dongfang University, Harbin, China.; 2 Dong Jiying Lecturer, College of Humanities, Heilongjiang Dongfang University, Harbin, China.; 3 Qu Qiumei Visiting Lecturer, Department of History & Pakistan Studies, University of the Punjab, Lahore, Pakistan.

**Keywords:** China, Pakistan, Traditional Medicine, Similarities, Modern Challenges

## Abstract

The use of medicinal plants within specific communities constitutes an important component of traditional cultural knowledge, passed down through generations and preserved as a valuable cultural heritage. Although extensive academic literature exists on the use of medicinal plants within individual cultures or specific ethnic groups, comparative studies across different countries and cultures remain limited. Over recent decades, the cross-cultural significance of medicinal plants among diverse ethnic communities worldwide has become increasingly apparent. This comparative approach is both practical and essential, as it highlights differences in the use of medicinal plants across cultures and creates opportunities for further research.

## INTRODUCTION

This study focuses on the Yunnan region of China, particularly the Dulong people of the Dulong River area, the Lisu people of the Nujiang region, cross-border ethnic groups (Hani, Dai, Lisu, Lahu, Wa, Jingpo, Blang, Achang, Nu, De’ang, and Dulong) in Yunnan, and ethnic communities in the Tibetan-Qiang-Yi Corridor (Zhuang, Bai, Lahu, Jingpo, Dulong, Miao, Hani, Wa, Pumi, Menba, Yi, Dai, Naxi, Achang, Tibetan, Lisu, Qiang, and Nu). These groups are compared with the indigenous communities of the Momand District in the former Federally Administered Tribal Areas (FATA) of northern Pakistan, the tribal populations of the Tehr-e-Suleiman Range, and the Amchies and Sanyasis.

Over the past few decades, the cross-cultural significance of medicinal plants among diverse ethnic groups worldwide has gained increasing recognition. Comparative research of this nature is both practical and important, as it helps identify similarities and differences in the use of medicinal plants between Yunnan, China’s southwestern frontier, and northern Pakistan, while also opening new avenues for future research.

## METHODOLOGY

Although quantitative research on this subject is abundant, qualitative studies remain limited. Therefore, this study primarily relies on a literature review, including ethnobotanical research, historical medical texts, and regional surveys of medicinal plants. A comparative analytical approach is employed to examine ethnobotanical methods, cross-cultural analytical frameworks, disease classification systems, and the uses of medicinal plants.

**Similarities Between Traditional Chinese**

**Medicine and Traditional Pakistani Medicine**

### Traditional Mountain Medicine Systems:

The regions under study share similar geographical characteristics, including rugged mountains, deep valleys, and extensive ravine systems, resulting in geographical isolation and limited accessibility.

The Dulong people of the Dulong River region in Yunnan inhabit a particularly isolated environment. Historically, they remained in a primitive stage of development, “recording events by carving wood and tying knots, and using birdsong and blooming flowers to mark the seasons.” Due to underdeveloped economic and cultural systems, limited healthcare infrastructure, and a shortage of trained medical personnel and medicines, research on traditional Dulong medicine remains scarce.¹

Similarly, the Lisu people of Yunnan’s Nujiang region have long lived in rugged mountainous terrain. Although this environment restricted communication with the outside world, it also provided favourable conditions for the growth of medicinal plants and animals. Through prolonged interaction with nature and disease, the Lisu accumulated substantial traditional medical knowledge.²

Most of Yunnan’s 11 unique ethnic groups inhabit economically underdeveloped mountainous regions with poor healthcare facilities. In these “frontier, mountainous, and impoverished” areas, traditional ethnic medicine has historically served as the primary means of disease prevention and treatment.³ Likewise, before the founding of the People’s Republic of China, the Tibetan-Qiang-Yi Corridor remained largely isolated due to poor transportation networks, low economic productivity, and inadequate healthcare facilities.^4^

Similarly, Pakistan’s Suleiman Mountain region is geographically remote and isolated. Medical resources among tribal communities are limited; however, local populations have developed extensive medical knowledge through practical experience and healing traditions passed down across generations. Local residents commonly rely on both traditional and modern biomedical treatments, often combining the two systems.^5^

The Momand District derives its name from the Momand tribe inhabiting the region. Characterized by rugged mountains and barren slopes, the district faces widespread poverty and marked socioeconomic disparities among indigenous communities.

### Oral Transmission of Medical Knowledge

Written literature on Dulong medicine remains limited, and much of its traditional knowledge has not been systematically documented. Instead, medical practices have been transmitted orally through hands-on instruction and the healing experiences of shamans.^6^

Although the Lisu possess a written language, their medical knowledge continues to be transmitted orally, resulting in a lack of written medical records or formal texts.³ Similarly, the traditional medical knowledge of the Lahu people has been preserved orally without a documented theoretical system.³ The Wa people, lacking a written language, also rely on oral transmission and folk practices to preserve traditional medicine.³

Before modernization, the De’ang people had no formally trained physicians and relied primarily on natural remedies, particularly medicinal herbs available near their villages. Diseases were generally named according to symptoms or affected body parts, while medicines were categorized according to the ailments they treated.³

The Nu and Dulong peoples of the Nujiang region, who inhabit deep mountain gorges with limited external contact, developed traditional medical systems based on ancestral experience. In the absence of a written language, medical knowledge was transmitted through oral teaching and practical guidance from one generation to the next. Over time, this knowledge evolved through continual refinement and practical application.³

Similarly, the Jingpo, Blang, Achang, Nu, and Dulong peoples accumulated extensive traditional medical knowledge through their experiences in combating disease. Although undocumented, this knowledge has survived through oral transmission.³

Ancient medical traditions in the Tibetan-Qiang-Yi Corridor may broadly be divided into three categories. The first includes ethnic groups with written languages and well-developed medical systems, such as the Tibetan, Yi, and Naxi peoples, who possess medical texts and theoretical frameworks. The second category includes groups with limited or fragmented written medical records, often documented in pictorial forms or other languages, such as the Qiang, Jingpo, and Bai peoples. The third category comprises ethnic groups lacking a written language, whose medical traditions are preserved orally through folklore, epics, and songs.^7^

In Pakistan, medicinal plant knowledge has similarly been transmitted orally for generations, particularly in tribal and rural communities where healing practices are deeply rooted in lived experience.^8^ In the Momand District of the former FATA region, traditional herbal knowledge is predominantly passed down through oral tradition.^9^

These observations reveal notable similarities between the cross-border ethnic regions of Southwestern China and the tribal mountainous communities of northern Pakistan in the transmission of traditional medical knowledge. In both regions, medical practices are largely undocumented and preserved through oral traditions, practical experience, and intergenerational learning within families, tribes, and among local healers. Disease treatment is deeply connected to cultural and religious traditions, while practical therapeutic knowledge remains central to healthcare practices. However, this mode of transmission faces a significant risk of decline and discontinuity.

### Psychotherapy and Spiritual Care

The religious and cultural traditions of the Dulong people, including festivals and rituals, are integral to their traditional medicine. Primitive religious beliefs concerning souls, spirits, and shamans underpin Dulong medical concepts and therapeutic practices.^6^ Illnesses such as “Nanmusha” are believed to be treated by mythical healers known as “Nanmu,” while shamans employ religious rituals alongside herbal remedies and practical therapeutic interventions.³

Historically, Lisu healers combined medicine with spiritual practices while simultaneously working as farmers, as no full-time medical practitioners existed. Religion and folk medicine continue to play an important role in preserving the physical and mental well-being of the Lisu people.³

Likewise, in the Tibetan-Qiang-Yi Corridor, harsh environmental conditions fostered a strong reliance on religious beliefs and spiritual healing. People often turned to religious figures, particularly lamas, for healthcare and spiritual support.^10^

Among the tribes of the Tehr-e-Suleiman Mountains in Pakistan, healing practices incorporate spiritual approaches, particularly for illnesses believed to be caused by jinn or spiritual disturbances. Conditions involving psychological distress, fear, or trauma are commonly treated through amulets, blessings, and prayers, often administered by local spiritual healers known as *Aamels*.

Similarly, communities in the Momand District maintain strong cultural beliefs in herbal remedies prepared by traditional healers, locally known as *Hakims*. Consequently, the integration of herbal medicine, religious beliefs, and tribal traditions remains a defining feature of healthcare practices in the region.

These findings suggest that, in both Yunnan and northern Pakistan, illness is often viewed not merely as a physiological condition but also as a manifestation of spiritual or supernatural imbalance. Traditional medical systems in both regions demonstrate a shared reliance on concepts linking humanity, nature, and spirituality. Although varying in degree, ethnic communities in both countries incorporate herbal and modern medical practices while adapting them to local cultural contexts. Through generations of experience, these communities have developed practical therapeutic approaches using locally available medicinal plants and food-based remedies to manage common diseases.

### Historical Role of Medicinal Plants

The concept of “food as medicine” is one of the primary approaches used by the Dulong people to treat illnesses and remains a key component of Dulong traditional medicine. According to historical records, the Dulong people collect more than one hundred species of medicinal plants. Among these, cordyceps, gastrodia, coptis, and fritillary bulb possess high economic value and were among the earliest medicinal herbs traded with the outside world. The collection of gastrodia, coptis, and fritillary bulb also constitutes an important source of livelihood for the Dulong community.^11^ In the Dulong River region, traditional healers use locally available herbal medicines to treat fractures, bruises, sprains, rheumatic disorders, abdominal pain, colds, hepatitis, and other ailments.^2^

In Nujiang Prefecture, a total of 689 medicinal plant species (including varieties and subspecies), representing 502 genera and 166 families, have been identified, along with 32 commonly used medicinal animal species.^12^ The traditional medicine practiced by Yunnan’s 11 cross-border ethnic groups reflects the health and wellness traditions of these minority communities. Its therapeutic practices are closely linked to the living environment, modes of production, and daily lifestyles of these groups, making it highly relevant to their everyday needs and widely accepted by local populations.^3^ The *Dictionary of Chinese Ethnic Medicines* records more than 5,000 ethnic medicines from the “Tibetan-Qiang-Yi Corridor” region. Herbal medicines constitute the majority of remedies used by ethnic groups in this region, followed by animal-based medicines, whereas mineral-based medicines are comparatively rare.^13^

Although several public health clinics in the Moman District provide limited healthcare services, residents living in remote mountainous areas often have little or no access to Western medicine. Consequently, local communities generally prefer traditional herbal remedies and place strong trust in herbal formulations prepared by traditional healers, locally known as “Hakim.”^14^ In northern Pakistan, *Amchies* and *Sanyasis* practice herbal medicine as part of Ayurvedic traditions. Unani medicine remains the dominant traditional medical system in Pakistan, and the use of medicinal plants for home remedies is widespread. There is also considerable public interest in homeopathic medicine.^15^ Similarly, residents of the Tahr-e-Suleiman Mountains rely on both traditional and biomedical treatments, often combining the two approaches.^5^

The literature suggests that the use of medicinal plants in the ethnic regions of Yunnan, China, and northern Pakistan is rooted in practical experience and shaped by geographical isolation, limited access to urban healthcare facilities, and economic constraints.

### Modern Challenges

Much of the knowledge and essence of Dulong medicine have not been systematically documented or preserved in a timely manner.^6^ Similarly, the Lisu people’s medicinal practices and knowledge of traditional herbal medicine have been transmitted orally from generation to generation and continue to evolve; however, without proper preservation, this knowledge is at risk of disappearing^2^.

The Nu people of the Nujiang region inhabit remote mountain gorges, where communication with the outside world has historically been limited. Over centuries, they developed a unique system of traditional medicine based on accumulated ancestral knowledge. Because the Nu people lacked a written language, medical knowledge was transmitted exclusively through oral instruction and practical training from one generation to the next. This intergenerational transfer became an essential component of traditional education for survival and continuity, while practical experience continually refined and expanded medical knowledge.^3^

The preservation and development of ethnic medicine in the “Tibetan-Qiang-Yi Corridor” are currently facing a serious crisis. Traditional transmission systems have weakened due to insufficient recognition, inadequate documentation and compilation of ancient medical texts, and policy restrictions limiting the “living” transmission of folk medicine.^13^ In some settings, Western medicine and traditional medicine are regarded as opposing systems, resulting in strict regulation of traditional practices, which are often labeled “anti-scientific.” Consequently, practitioners may face legal or professional penalties. Furthermore, variations in local names for medicinal herbs and the lack of regulatory oversight among unlicensed practitioners have contributed to a decline in the credibility and social acceptance of herbal medicine.^16^

Likewise, the traditional medical knowledge of tribes in the Suleiman Mountains has been significantly affected by cultural assimilation and modernization. Unless documented and preserved, this valuable heritage may be lost to future generations.^5^ In Indigenous communities of the Mormande Special Administrative Region, herbal medicine knowledge is also primarily transmitted orally, increasing the risk of gradual loss over time and posing a major challenge to its preservation.^14^

In summary, both China and Pakistan face similar challenges regarding the preservation of traditional medicine. Modernization, the weakening of oral traditions, inadequate cultural recognition, the growing influence of Western medicine, and the lack of trained successors have contributed to the gradual decline of traditional medical systems. Therefore, comprehensive efforts are needed to improve the quality and standards of traditional medicine research in both countries. Dedicated funding mechanisms should be established to support the protection and development of Dulong traditional medicine, while local communities should be encouraged to develop sustainable traditional medicine industries.

The governments of both countries should strengthen cultural exchange and collaboration in traditional medicine and formulate supportive policies for existing practitioners, including legal recognition of their practice to facilitate the preservation and transmission of traditional medical knowledge. Although local governments have initiated efforts to encourage the cultivation of selected medicinal species, most valuable medicinal herbs have yet to be cultivated on a large scale or industrialized. Greater efforts are required to conserve wild medicinal plants and establish medicinal herb cultivation bases for Chinese and ethnic medicines.

Public awareness and understanding of traditional medicine should also be enhanced through scientific outreach, educational programs, and vocational training. Such initiatives can help communities develop a scientific appreciation of their ethnic medical traditions while promoting greater collaboration and exchange between China and Pakistan. Efforts to preserve and promote ethnic medical culture may also strengthen cultural identity, improve patient adherence to treatment, and contribute to the overall health and well-being of communities.

## CONCLUSIONS

The urgent documentation and systematic compilation of traditional medical knowledge in China and Pakistan are essential for its preservation and transmission. Due to the scarcity of relevant literature and the predominance of oral transmission, much of the valuable knowledge embedded in these medical traditions has not been adequately recorded or safeguarded.

Folklore and cultural customs remain important sources for understanding traditional medical practices in both countries. Further research into the influence of folklore and customs on medical philosophies, as well as the identification of shared patterns and underlying connections, represents an important area for future study. Moreover, examining the cultural similarities and differences between Yunnan in southwestern China and northern Pakistan offers promising opportunities for advancing comparative research in traditional medicine.
